# Serotonergic Signaling Governs *Caenorhabditis elegans* Sensory Response to Conflicting Chemosensory Stimuli

**DOI:** 10.1523/ENEURO.0127-25.2025

**Published:** 2025-07-17

**Authors:** Caroline S. Muirhead, Sophia Guerra, Bennett W. Fox, Frank C. Schroeder, Jagan Srinivasan

**Affiliations:** ^1^ Department of Biology and Biotechnology, Worcester Polytechnic Institute, Worcester, Massachusetts 01609; ^2^ Northeastern University, Boston, Massachusetts 02115; ^3^ Department of Chemistry and Chemical Biology, Cornell University, Ithaca, New York 14853

**Keywords:** ascarosides, food, inhibition, neural circuits, neurons, serotonin

## Abstract

Neural circuits that consolidate sensory cues are essential for neurological functioning. Neural circuits that perform sensory integration can vary greatly because the sensory processing regions of the brain employ various neural motifs. Here, we investigate a neural circuit that mediates the response to conflicting stimuli in *Caenorhabditis elegans*. We concurrently expose animals to an aversive dispersal pheromone, osas#9, and an attractive bacterial extract. While worms usually avoid osas#9 alone, they suppress this avoidance behavior in the presence of a bacterial extract. Loss-of-function mutants and cell-specific rescues reveal that signaling from the ADF and NSM neurons is essential for bacterial extract-induced osas#9 avoidance attenuation. The inhibitory serotonin receptor, MOD-1, which is widely expressed on interneurons and motor neurons, is required for this sensory integration, suggesting that serotonin acts in an inhibitory manner. By performing calcium imaging on the ADF neurons in synaptic signaling (*unc-13*) and peptidergic (*unc-31*) signaling mutant backgrounds, we show that the ADF neurons require input from other neurons to respond to food extracts. We reveal a cue integration neural circuit in which serotonergic signaling and sensory neurons silence an aversive neural signal.

## Significance Statement

Animals use sensory cues to make behavioral choices and sometimes, these cues convey opposite information. The nervous system consolidates competing sensory cues to create a coherent response to external stimuli. The neural circuits that govern this process are important and still largely unknown. We use *Caenorhabditis elegans*, a soil-dwelling nematode, to uncover a neural circuit governing the consolidation of competing cues by concurrently exposing worms to positive and negative stimuli. We find that the neurotransmitter serotonin can suppress aversive neural signals created by negative stimuli. These results show the important neurological role that serotonin plays in modulating neural signals.

## Introduction

The nervous system's ability to consolidate sensory input is essential (Wallace et al., 2020). The nervous system constantly receives sensory input from multiple sensory modalities (Stein et al., 2014; Yau et al., 2015). Additionally, the nervous system must consolidate competing sensory information from the same modality (Blake and Logothetis, 2002; Cohen Kadosh et al., 2008). Impairment in sensory integration is associated with a variety of neurological disorders, including autism spectrum disorder, attention deficit hyperactivity disorder, and schizophrenia (Ghanizadeh, 2011; Koziol et al., 2011; Tseng et al., 2015). Across the animal kingdom, neural circuits that perform sensory integration tasks generally use coordinated excitation and inhibition (Yan, 2002; Wang, 2008; Miller et al., 2011; Wu and Guo, 2011; Chadderton et al., 2014; Herd et al., 2021). In the human brain sensory, information processing is complex, and therefore, these circuits can vary (Bertrand and Thomas, 2004; Chadderton et al., 2014; Leighton and Lohmann, 2016). In fact, models of sensory integration in mammals are only made to apply to local neural circuits—not the whole mammal brain (Sabes, 2011).

*Caenorhabditis elegans* are an ideal model for studying small neural circuits that integrate sensory information. They are both simple and amenable to experimentation (Chalfie and Jorgensen, 1998). *C. elegans* hermaphrodites contain precisely 302 fully mapped neurons (White et al., 1986). Additionally, *C. elegans* display robust behavioral outputs in response to various stimuli (de Bono and Maricq, 2005), and since they are transparent, observing fluorescent protein expression and imaging experiments are straightforward (Félix and Braendle, 2010). Together, these attributes allow for manipulation of specific neurons, receptors, or neural connections and observation of the corresponding behaviors, making it possible to understand how individual cells act within a neural circuit.

Since *C. elegans* lack complex sensory organs, they communicate via olfaction (Bargmann, 2006). Pheromones released by *C. elegans*, ascarosides, are used to communicate a wide variety of information (Ludewig and Schroeder, 2013; Muirhead and Srinivasan, 2020); worms will attract mates (Izrayelit et al., 2012; Chute and Srinivasan, 2014), communicate food levels (Artyukhin et al., 2013), and promote entry into dauer phase using these secreted pheromones (Albert et al., 1981; Edison, 2009). Here, we utilize a specific ascaroside, osas#9, to study sensory integration. osas#9 is produced primarily by food-deprived L1 animals (Artyukhin et al., 2013). Given that the biosynthesis of osas#9 is dependent on starvation, it is unsurprising that it functions as a dispersal cue and communicates lack of food in the environment (Chute et al., 2019). Structurally, osas#9 is unique among the ascarosides because it incorporates the neurotransmitter octopamine (Artyukhin et al., 2013; Chute et al., 2019). Previous work with osas#9 has determined that it is sensed primarily by the nociceptive ASH sensory neuron via a GPCR, TYRA-2 (Chute et al., 2019).

Here, we concurrently exposed animals to osas#9 (an aversive cue) with an opposite cue (an attractive bacterial extract) to determine how the nervous system reconciles competing sensory cues. We find that serotonergic signaling from NSM neurons and signaling from ADF sensory neurons is essential for the integration of the food cue into the nervous system. We also show that the ADF neurons act with other neurons in order to achieve this integration.

## Materials and Methods

### Worm maintenance

Animals were maintained on nematode growth media (NGM) plates at 20°C. Animals were fed OP50 *Escherichia coli* (Brenner, 1974); young adult animals were transferred to new plates to avoid starvation. Worm strains used are shown in Table S1.

### Avoidance assay

The avoidance assay was performed as previously described (Hilliard et al., 2002). Animals were transferred onto unseeded NGM plates. Animals were starved 1 h prior to behavioral testing. A copper ring was placed on the NGM plate to minimize animals leaving the plate.

The avoidance assay was performed by placing a small (roughly 5 nl) drop of stimulus dissolved in diH_2_O or a solvent control on the tail of a forward moving animal. The solvent control consists of the solvent for the stimulus; we used diH_2_O with 1% ethanol because the both osas#9 and osas#9 + extract mixtures contain 1% ethanol. Drops were created using capillary tubing. Once the liquid encountered the animal's head via capillary action, the animal's response would be scored as avoidance or nonavoidance. Drops were not large enough to cause thrashing behavior in animals (Movies 1, 2). Given that sensory neurons detecting repellents are located in the animal's head, animals will reverse when encountering a negative stimuli (Goodman and Sengupta, 2019). If within 5 s of encountering a drop, the worm showed at least two body bend reversals or a 180° omega turn, the behavior was scored as avoidance (Gray et al., 2005; Movie 1). Other responses (continued forward movement, stopping without a reversal, or one backward body bend) were scored as nonavoidance (Movie 2). Animals were scored blindly with respect to genotype. Eight to 15 individual worms were tested per plate and a minimum of six plates were tested over a minimum of three different days. For each individual plate, an avoidance index was calculated. Avoidance index was calculated as number of worms that avoid a stimulus divided by the total number of worms tested per plate.

**Movie 1.** Avoidance drop assay with an approximately 5 nL solution on the worm's tail; absence of avoidance is indicated by continued movement. [View online]

**Movie 2.** Avoidance drop assay with approximately 5 nL solution on the worm's tail; the worm reverses with two body bends within 4 seconds of exposure, indicating avoidance of the chemical. [View online]

### Calcium imaging

Prior to imaging, worms were starved for 1 h on NGM plates so that they would have the same internal state during imaging and behavior.

Calcium imaging was performed in a microfluidic device previously described by Reilly et al. (2017). Animals were immobilized in a PDMS chip. The nose of the worm was exposed to either solvent control or stimulus liquid. All stimuli were dissolved in S. basal buffer with 1 mM tetramisole as a paralytic to assist in immobilizing the animals. Solvent and flow controls were 1 mM tetramisole S. basal with 0.1 and 0.2 µg/ml of fluorescein, respectively.

During a trial, an animal was exposed to the solvent control for 5 s. Then, it was exposed to a pulse of stimulus for 30 s. Finally, it was exposed to solvent control for another 15 s.

Intensity of pixel fluorescence in neurons was analyzed using Fiji. The neuron was morphologically identified and traced as the ROI. The average fluorescence intensity was calculated for each frame in the ROI. The same size and shape ROI was placed on an area of the worm head without the neuron, and then average fluorescence intensity was calculated for each frame in the background ROI. A typical ROI is ∼1,700 pixels. Δ*F* was calculated by subtracting the fluorescence intensity of the background ROI from the fluorescence intensity of the neuron body ROI. *F*_o_ is the average Δ*F* for 10 frames prior to stimulation. To obtain %Δ *F*/*F*_o_, one was subtracted from Δ*F*/*F*_o_, then that quantity was multiplied by 100. Max peak Δ*F*/*F*_o_ refers to the largest Δ*F*/*F*_o_ value detected.

We scored response and nonresponse to stimulation using the following criteria: if an animal's maximum response during the stimulation period was equal to or greater than 3 times the maximum response during the prestimulation period, we counted that animal as a responder. Otherwise, the animal was recorded as a nonresponder.

### Microscopy

Animals were mounted on a 2% noble agar pad and immobilized using 1 µl of 1 M sodium azide. Images were obtained using a Zeiss LSM510 Meta inverted confocal microscope. Then, 1-µm-thick *z*-stacks slices were used to capture the whole head region of the animal. Images were analyzed using ImageJ software.

### Preparation of bacterial extract

Ten milliliter of methanol were added to one gram of freeze-dried *E. coli* OP50 powder (InVivo Biosystems, formerly NemaMetrix, OP-50-31772) in a 50 ml conical tube and then sonicated for 5 min (2 s on/off pulse cycle at 90 A) using a Qsonica Q700 Ultrasonic Processor with a water bath cup horn adaptor (Qsonica 431C2). Following sonication, the conical tube was centrifuged (3,000 × *g*, 22°C, 5 min) and the resulting clarified supernatant transferred to a clean pear flask. This process was repeated twice more, and each time the clarified supernatant was transferred to the same pear flask. After the third extraction, the insoluble material was washed with 10 ml methanol and vortexed for 10 s and then subject to centrifugation (3,000 × *g*, 22°C, 5 min) and the resulting clarified supernatant transferred to the same pear flask. One additional methanol wash was performed, and the supernatant was combined in the same pear flask, resulting in ∼50 ml methanol. An aliquot of the methanol extract was transferred to a clean 8 ml glass vial and concentrated to dryness in an SC250EXP SpeedVac Concentrator coupled to an RVT5105 Refrigerated Vapor Trap (Thermo Scientific). The resulting powder (14.6 mg) was resuspended in 1 ml methanol.

### Statistical analysis

Statistical analyses were performed in GraphPad Prism. When multiple comparisons were being made, the Shapiro–Wilk normalcy test was performed to determine if data were normally distributed. If data were normally distributed, an ordinary one-way ANOVA was performed followed by Sidak's multiple comparisons. If data were not normally distributed, then a Kruskal–Wallis test was performed followed by Dunn's multiple comparisons. When comparing an animal to itself (pre- and postimaging stimulus), a Shapiro–Wilk normalcy test was performed. If the data were not normally distributed, a two-tailed Wilcoxon test was performed to find differences pre- and poststimulation. Otherwise, a two-tailed paired *t* test was performed. To compare the number of animals responding to stimulation during calcium imaging and the number of animals not responding to stimulation during calcium imaging, we performed Fisher's exact test.

## Results

### Food-deprived *C. elegans* suppress osas#9 avoidance in the presence of a food cue

**Figure 1.**
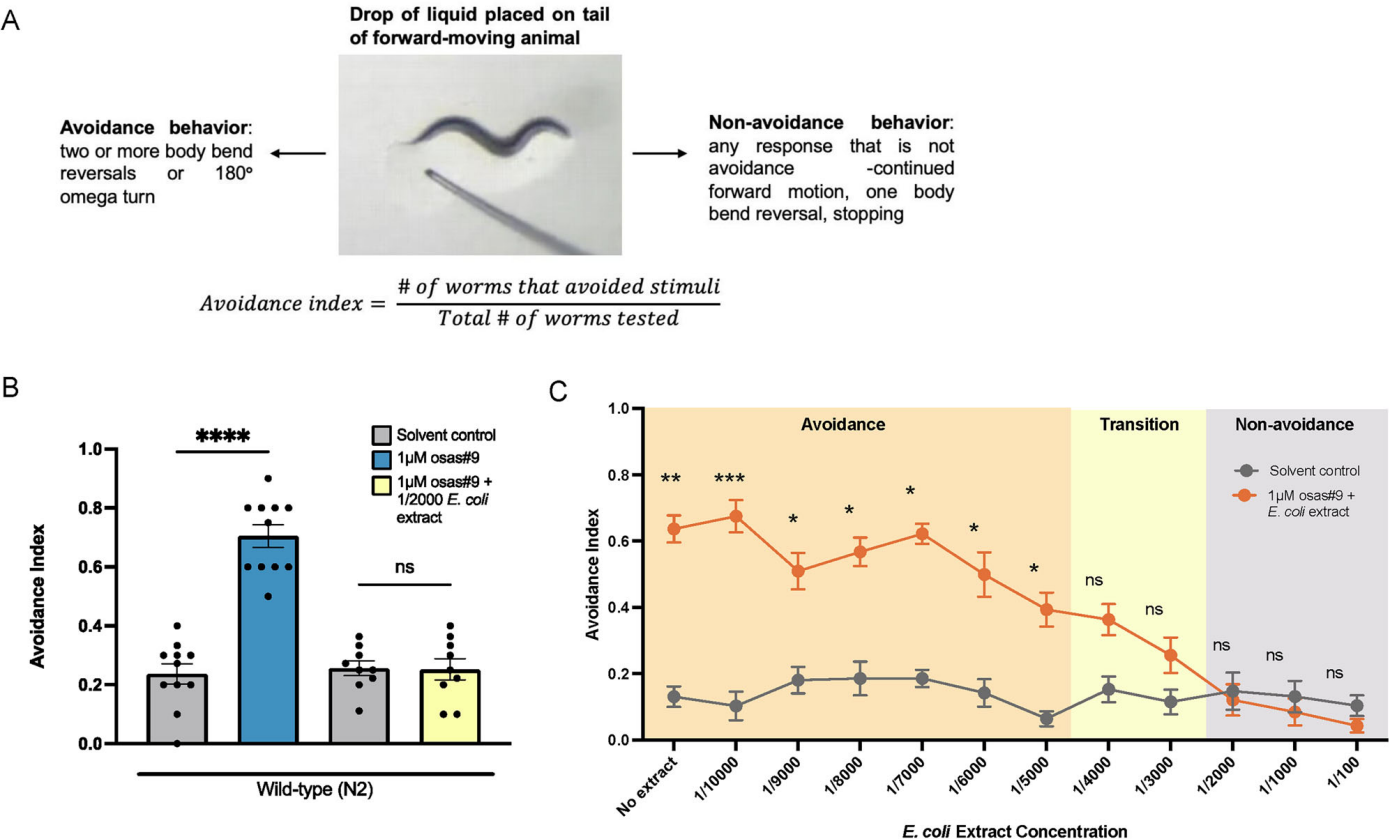
Food cues attenuate osas#9 avoidance in food-deprived animals. ***A***, Schematic of avoidance index assay. ***B***, Food-deprived worms will no longer avoid 1 µM osas#9 when *E. coli* extract is present in the stimulus mixture. *n *≥ 9. Shapiro–Wilk normalcy test followed by ordinary one-way ANOVA w/Sidak's multiple comparisons. ***C***, Food extract-induced osas#9 avoidance attenuation is dependent on extract concentration. When high concentrations (1/100–1/2,000) of extract are present in the osas#9 mixture, avoidance is strongly attenuated. Intermediate concentrations of food extract (1/3,000–1/4,000) still suppress avoidance but there is a trend of increasing avoidance. Low concentrations of food extract (1/5,000–1/10,000) present in the mixture are not sufficient to attenuate osas#9 avoidance. *n *≥ 8. Shapiro–Wilk normalcy test followed by ordinary one-way ANOVA w/Sidak's multiple comparisons. Error bars are SEM. *p < 0.05, **p < 0.01, ***p < 0.001, ****p < 0.0001.

Previous work has demonstrated that food-deprived worms avoid the pheromone osas#9, whereas fed animals show no aversive behavior (Artyukhin et al., 2013; Chute et al., 2019; Extended Data Fig. 1-1*A*). Because of the myriad changes that occur between the fasted and fed states (Baugh and Hu, 2020), we wanted to create a situation in which worms must choose between two competing stimuli without providing a nutritive source. To do this, we concurrently exposed starved animals to both osas#9 and an extract of the bacterial food (*E. coli*) using the avoidance assay (Fig. 1*A*) and observed that worms no longer avoided osas#9 in the presence of *E. coli* extract (Fig. 1*B*). Notably, worms will not avoid *E. coli* extract alone (Extended Data Fig. 1-1*B*). Since sensory processing is typically dependent on cue strength, we tested avoidance behavior to osas#9 mixed with varying food extract concentrations and saw that food extract-dependent osas#9 avoidance attenuation is concentration dependent (Fig. 1*C*). We observed that high concentrations of food extract strongly suppressed avoidance to osas#9 (1/10–1/2,000; Fig. 1*C*). Conversely, worms avoided the osas#9 *E. coli* extract mixture at low concentrations, (1/5,000–1/10,000; Fig. 1*C*). We also observed that at intermediate concentrations of extract (1/3,000–1/4,000), worms did not avoid the mixture significantly more than the solvent control, but there was a trend of increased avoidance (Fig. 1*C*).

10.1523/ENEURO.0127-25.2025.f1-1 Figure 1-1 a. Animals will begin avoiding 1  µM osas#9 30 minutes after food removal. Prior to 30 minutes, avoidance index to 1  µM osas#9 is no different than the solvent control. The avoidance index appear to plateau after 30 minutes of food removal. n >= 7.b. Animals do not avoid *E. coli* extract alone. Download Figure 1-1, TIF file.

**Figure 2.**
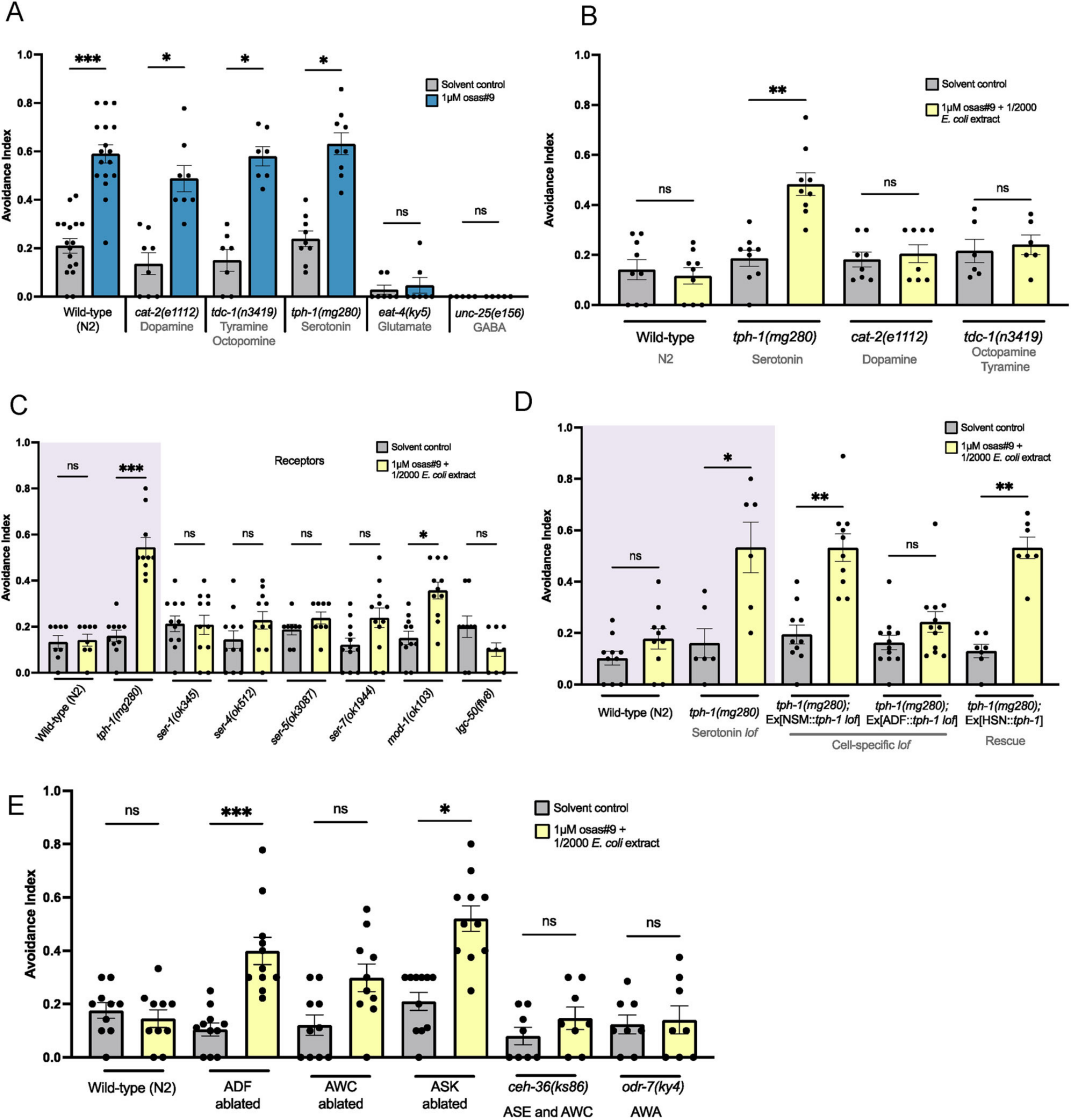
Serotonergic signaling is required for food-dependent osas#9 avoidance attenuation. ***A***, Glutamate and GABA are required for osas#9 avoidance. A screen of neurotransmitter mutants showed that *eat-4(ky5)* mutants and *unc-25(e156)* do not avoid osas#9. *n *≥ 5. Shapiro–Wilk normalcy test followed by Kruskal–Wallis test with Dunn's multiple comparisons. ***B***, A screen of neurotransmitter mutants showed that *tph-1(mg280)* mutants avoid the osas#9 and food extract mixture. *n *≥ 6. Shapiro–Wilk normalcy test followed by Kruskal–Wallis test with Dunn's multiple comparisons. ***C***, *mod-1(ok103)* mutants avoid osas#9 when mixed with food extract. *n *≥ 8. Shapiro–Wilk normalcy test followed by Kruskal–Wallis test with Dunn's multiple comparisons. ***D***, Removing *tph-1* function in the NSM neurons but not the ADF neurons resulted in osas#9 + extract avoidance. Rescuing *tph-1* in the HSN neurons resulted in no behavioral rescue. *n *≥ 6. Shapiro–Wilk normalcy test followed by Kruskal–Wallis test with Dunn's multiple comparisons. ***E***, Ablation of the ADF and ASK neurons results in avoidance to osas#9 + food extract. Ablation of the AWC neurons has no effect on avoidance attenuation behavior. *ceh-36(ks86)* and *odr-7(ky4)* show normal avoidance attenuation. *n *≥ 8. Shapiro–Wilk normalcy test followed by Kruskal–Wallis test with Dunn's multiple comparisons. Error bars are SEM. *p < 0.05, **p < 0.01, ***p < 0.001, ****p < 0.0001.

10.1523/ENEURO.0127-25.2025.f1-2 Figure 1-2 a. Ablation of ADF, ASK, or AWC neurons does not impact osas#9 avoidance; *ceh-36* and *odr-7* animals avoid osas#9.b. Animals containing *tph-1*::GFP constructs showed *tph-1* expression in the ADF, HSN, and NSM neurons. 63X magnification; oil objective. Download Figure 1-2, TIF file.

10.1523/ENEURO.0127-25.2025.t1-1 Table 1-1 List of strains used in this manuscript. Download Table 1-1, DOCX file.

### Food-dependent osas#9 avoidance attenuation is dependent on serotonin signaling and sensory neurons

We wanted to know which neurotransmitters, if any, are involved in modulating the nervous system's suppression of osas#9 avoidance in the presence of food extract. We tested *tph-1*, *cat-2*, and *tdc-1* mutants. *tph-1* encodes tryptophan hydroxylase, an enzyme required for the neuronal biosynthesis of serotonin (Sze et al., 2000; Yu et al., 2023). *cat-2* encodes an enzyme required for dopamine synthesis, tyrosine hydroxylase (Lints and Emmons, 1999). Finally, *tdc-1* mutants lack both tyramine and octopamine (Alkema et al., 2005). We found that *tph-1* mutants, while they avoided osas#9 alone normally (Fig. 2*A*), continued to avoid osas#9 even when it was mixed with food extract (Fig. 2*B*). This result indicates that serotonin is required for the integration of food cues into the osas#9 avoidance neural circuit. We also tested mutants that are deficient in glutamatergic synaptic transmission (*eat-4*) (Lee et al., 1999) and GABA biosynthesis (*unc-25*; Jin et al., 1999), but these mutants did not avoid osas#9 alone normally, so those strains were excluded from our screen (Fig. 2*A*, Extended Data Table 1).

There are six known serotonin receptors in *C. elegans* (Chase and Koelle, 2007; Hapiak et al., 2009; Morud et al., 2021). To find out which serotonin receptor(s) were acting to attenuate osas#9 avoidance in the presence of *E. coli* extract, we tested mutants for all six serotonin receptors: *ser-1*, *ser-4*, *ser-5*, *ser-7*, *mod-1*, and *lgc-50*. The *ser* receptors are reported to be GPCRs (Carre-Pierrat et al., 2006; Chase and Koelle, 2007; Hapiak et al., 2009) whereas MOD-1 is a chloride channel (Ranganathan et al., 2000) and LGC-50 is a cation channel (Morud et al., 2021). We found that *mod-1* mutants avoided osas#9 mixed with food, indicating that this receptor is essential for the proper integration of the food cue (Fig. 2*C*). However, we also noticed that avoidance behavior in *mod-1* worms trended a little bit lower than avoidance behavior of *tph-1* mutants. To further investigate if any additional receptors are involved in avoidance behavior, it would be possible to test a *mod-1* double mutant with another receptor that trended lower in avoidance behavior.

In addition to investigating which serotonin receptors were acting to suppress osas#9 avoidance, we wanted to know which serotonin-releasing neuron(s) may be responsible for the avoidance suppression. There are three main neurons in *C. elegans* that express *tph-1*, and therefore, these are the three main neurons that produce serotonin: ADF, HSN, and NSM (Sze et al., 2000; Chang et al., 2006; Kullyev et al., 2010). There is also mild *tph-1* expression reported in the AIM and RIH neurons (Sze et al., 2000). We imaged a *tph-1*::GFP reporter strain and, consistent with previously published data, observed *tph-1* expression in the ADF, HSN, and NSM neurons (Extended Data Fig. 1-2). The HSNs are primarily involved in egg-laying behavior (Weinshenker et al., 1995; Shyn et al., 2003), while the NSM neurons are implicated in the bacterial-slowing response (Sawin et al., 2000) and to a lesser extent, pharyngeal pumping (Riddle et al., 1997; Li et al., 2012)—both food-related behaviors. The ADF neurons are unique in that they are the only serotonergic sensory neurons (Sze et al., 2000; Chang et al., 2006; Kullyev et al., 2010). Furthermore, the ADF neurons serve more diverse functions than other serotonergic neurons; they are involved in chemotaxis (Bargmann and Horvitz, 1991a), dauer entry (Bargmann and Horvitz, 1991b; Hu, 2005-2018), and sex-specific pheromone response (Fagan et al., 2018). To find out which neurons *tph-1* was acting in, we tested cell-specific rescues and cell-specific loss-of-function strains. Testing a *tph-1* rescue in the HSN neurons showed no rescue behavior, indicating that *tph-1* expression is not required in the HSN neurons to attenuate avoidance (Fig. 2*D*). Testing stains that removed *tph-1* function in the ADF and NSM neurons showed that *tph-1* expression in the NSM neurons is required for food-dependent avoidance attenuation behavior (Fig. 2*D*). The NSM neurons are not known to have cilia open to external stimuli (Axäng et al., 2008). Because of this, we believed that sensory neurons were also playing a role in avoidance attenuation via primary food extract sensation. We hypothesized that other neurons that are known to be involved in food sensation may be acting in the osas#9 avoidance attenuation neural circuit in concert with the NSM neurons. We tested ADF, ASK, and AWC neural ablation strains, in addition to *ceh-36* (defective sensation in ASE and AWC neurons; Koga and Ohshima, 2004), and *odr-7* (defective sensation in AWA neurons; Sengupta et al., 1994) mutants for their ability to suppress osas#9 avoidance in the presence of bacterial extract. All strains displayed normal avoidance to osas#9 alone (Extended Data Fig. 1-2*B*). We observed that worms avoided osas#9 in the presence of bacterial extract when the ADF neurons were ablated (Fig. 2*E*). In addition, we found that the ASK ablation strain avoided the osas#9-food mixture (Fig. 2*E*). Overall, these results show that serotonin signaling from the NSM neurons along with sensory neuron signaling is essential for canceling the osas#9 avoidance signal in the presence of food cues.

### Calcium transients increase in the ADF neurons upon *E. coli* extract exposure

**Figure 3.**
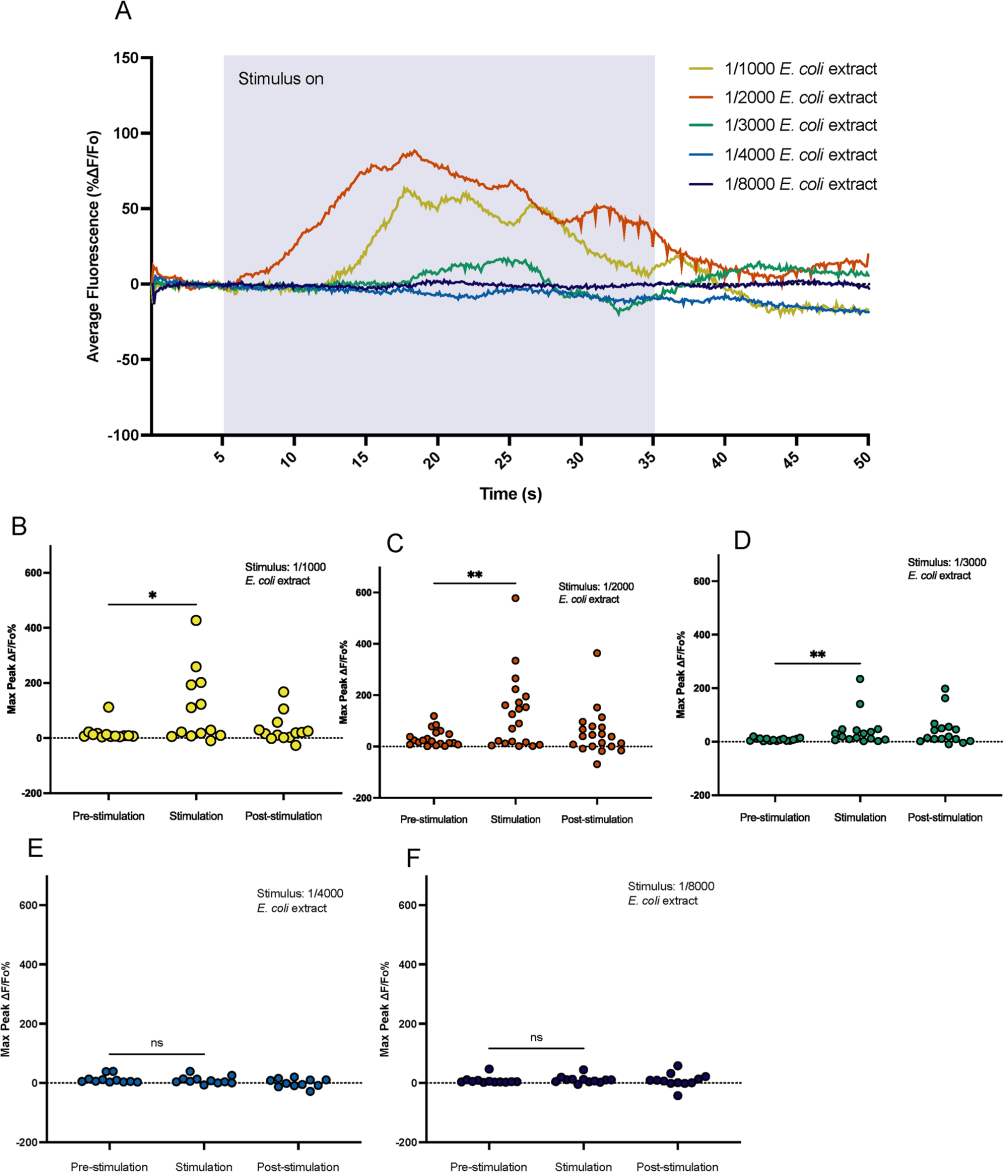
The ADF neuron responds to food cues. ***A***, Calcium dynamics of the ADF neuron in ADF::GCaMP animals upon exposure to different concentrations of *E. coli* extract. Different concentrations depicted in different colors. Error clouds surrounding trace of means are SEM. *n *≥ 11. ***B***, Maximum fluorescence intensity in the ADF neurons is significantly different after exposure to 1/1,000 food extract. *n* = 13. ***C***, Maximum fluorescence intensity in the ADF neurons is significantly different after exposure to 1/2,000 food extract. *n* = 20. ***D***, Maximum fluorescence intensity in the ADF neurons is significantly different after exposure to 1/3,000 food extract. *n* > 16. ***E***, There is no difference in maximum fluorescence intensity in the ADF neurons pre and post exposure to 1/4,000 food extract. *n* = 11. ***F***, There is no difference in maximum fluorescence intensity in the ADF neurons pre- and postexposure to 1/10,000 food extract. *n* = 12. *p < 0.05, **p < 0.01, ***p < 0.001, ****p < 0.0001. Shapiro–Wilk normalcy test followed by two-tailed Wilcoxon test pre stim versus stim.

Given that the ADF neurons are also serotonergic neurons, we wanted to better understand how the ADF neurons might be responding to food-derived chemicals; we performed calcium imaging. Transgenic worms expressing GCaMP in the ADF neurons under the *srh-142* promotor were loaded into microfluidic device (Reilly et al., 2017) and exposed to bacterial extract. We observed an increase in calcium transients in the ADF neurons upon stimulation with bacterial extract (Fig. 3*A*). At 1/1,000 and 1/2,000 concentrations of extract, we saw a strong increase in calcium transients (Fig. 3*A–C*; Extended Data Fig. 3-1*A,B*); this is consistent with the strong avoidance attenuation we observed at these concentrations of food extract (Fig. 1*C*). We wanted to know if intermediate concentrations of bacterial extract created a response in the ADF neuron, so we stimulated the neuron with 1/3,000 and 1/4,000 concentration of food extract (Fig. 3*A*,*D*,*E*; Extended Data Fig. 3-1*C,D*). We saw that at 1/3,000 concentration of food extract, the neuron showed a significant response (Fig. 3*D*); while at 1/4000, the neuron did not significantly respond (Fig. 3*E*). This result showed us that these intermediate concentrations appear to be on the lower end of where worms will detectably still respond to food extract, consistent with behavioral data that shows weaker avoidance attenuation (Fig. 1*C*). Finally, we hypothesized that low concentrations of bacterial extract, concentrations which were unable to attenuate osas#9 avoidance, would not produce any observable increase in calcium transients in the ADF neurons. To test this, we exposed animals to 1/8,000 food extract (a low concentration chosen from behavioral data) and saw that there was no observable neural response (Fig. 3*A*,*F*; Extended Data Fig. 3-1*E*). Notably, even among concentrations at which animals responded, we saw different response magnitudes. To quantify this, we compared the maximum peak responses during stimulation at each concentration to 1/2,000 *E. coli* extract responses (this concentration was chosen because it was used for behavioral data). We saw that the 1/2,000 extract responses did not differ from the 1/1,000 and 1/3,000 responses but that it was significantly greater than the 1/4,000 and 1/8,000 responses (Extended Data Fig. 3-1*F*).

10.1523/ENEURO.0127-25.2025.f3-1 Figure 3-1 Heatmaps of fluorescence intensity in the ADF neurons in ADF::GCaMP animals upon exposure to a.1/1000 *E. coli* extract, b. 1/2000 *E. coli* extract, c. 1/3000 *E. coli* extract, d. 1/4000 *E. coli* extract, e. 1/8000 *E. coli* extract. n >=11. f. Magnitude of maximum peak response during stimulation with each extract concentration. Shapiro-wilk normalcy test followed by ordinary one-way ANOVA w/ Sidak’s multiple comparisons. All concentrations compared to 1/2000 extract because this was the concentration used for behavioral assays. *p < 0.05, **p < 0.01, ***p < 0.001, ****p < 0.0001. Download Figure 3-1, TIF file.

10.1523/ENEURO.0127-25.2025.f3-2 Figure 3-2 The number of responding and non-responding animals during calcium imaging of the ADF neurons using different concentrations of *E. coli* extract as a stimulus. An animal was counted as a responder if its maximum response during stimulation was three times or greater than its maximum response during the pre-stimulation period. Fisher's exact test was performed to determine differences in response rate between conditions. P = 0.054. Download Figure 3-2, DOCX file.

Even with strong food stimulation, we observed that not all animals responded to *E. coli* extract. We quantified the number of animals responding during stimulation with 1/1,000 and 1/2,000 *E. coli* extract, roughly half of animals responded (Extended Data Table 2). This is likely due to natural response variation and because calcium sensors, like GCaMP, have limitations in their sensitivity and kinetics (Sun et al., 2013; Dana et al., 2019). Finally, the observed bimodal response could also be to left–right asymmetry in the ADF neurons, much like in the AWC neuron's response asymmetry (Troemel et al., 1999). However, this has not been established in the ADF neurons. Together, these imaging experiments indicate that the ADF neurons have increased calcium transients in response to food compounds when there is a sufficient amount of food, further supporting our behavioral data that osas#9 avoidance attenuation is dependent on food concentration.

### The ADF neurons require input from other neurons in order to respond to food extract

**Figure 4.**
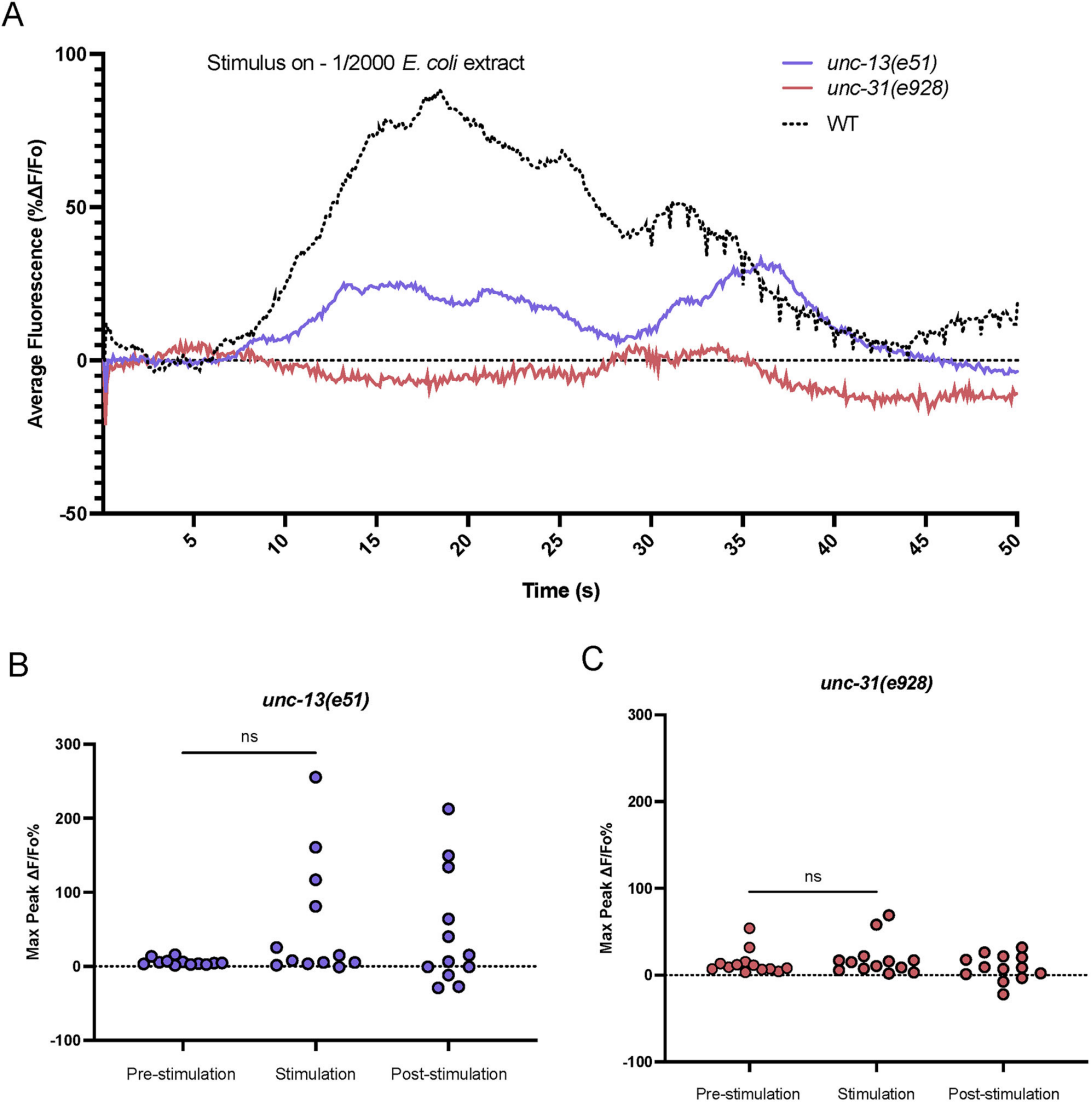
The ADF neurons require synaptic and peptidergic input for response to food extract. ***A***, Calcium dynamics of the ADF neurons in ADF::GCaMP animals in *unc-13(e51)* and *unc-31(e928)* backgrounds upon exposure to 1/2,000 *E. coli* extract. Error clouds surrounding trace of means are SEM. *n *≥ 12. Black dotted line depicts average trace of wild-type background calcium response. ***B***, There is no difference in maximum fluorescence intensity in the ADF neuron pre- and postexposure to 1/2,000 food extract in an *unc-13(e51)* background. *n* = 12. ***C***, There is no difference in maximum fluorescence intensity in the ADF neuron pre- and postexposure to 1/2,000 food extract in an *unc-31(e928)* background. *n* = 14. *p < 0.05, **p < 0.01, ***p < 0.001, ****p < 0.0001. Shapiro–Wilk normalcy test followed by two-tailed Wilcoxon test pre stim versus stim.

Since we found that the ADF neurons are essential for integrating food signals into the nervous system, we wanted to find out if the ADF neurons require input from other cells to integrate the food signal. To answer this question, we crossed the ADF imaging strain into *unc-13* and *unc-31* mutant backgrounds. These mutants are deficient in synaptic and peptidergic signaling, respectively (Richmond et al., 1999; Speese et al., 2007). If the ADF neurons still respond to food in the absence of synaptic and peptidergic communication, this will lead us to believe that the ADF neurons directly sense *E. coli* extract. However, if the ADF neurons requires synaptic or peptidergic signaling to respond, we will hypothesize that the ADF neurons function downstream of a different sensory neuron. Upon exposing either *unc-13* or *unc-31* ADF imaging strains to food extract, we saw that there was no significant increase in fluorescence in the ADF neuron upon food exposure in either mutant (Fig. 4*A–C*; Extended Data Fig. 4-1*A,B*). However, using a 3× response criteria showed that 58% of *unc-13* mutants responded, while only 8% of *unc-31* mutants showed response to stimulation, which was statistically different (Extended Data Table 3-2). These data suggest some type of response for mutants that lack synaptic signaling, indicating that the ADF neurons may have some role in the absence of synaptic communication. However, these data also show that the ADF neurons require both synaptic and peptidergic input from other neurons in order to fully respond to food extract.

10.1523/ENEURO.0127-25.2025.f4-1 Figure 4-1 Heatmaps of fluorescence intensity in the ADF neuron in ADF::GCaMP animals upon exposure to 1/2000 *E. coli* extract in a. an *unc-13(e51)* background and b. an *unc-31(e928)* background. Download Figure 4-1, TIF file.

10.1523/ENEURO.0127-25.2025.f4-2 Figure 4-2 The number of *unc-13* and *unc-31* responding and non-responding animals during calcium imaging of the ADF neurons using 1/2000 *E. coli* extract as a stimulus. An animal was counted as a responder if its maximum response during stimulation was three times or greater than its maximum response during the pre-stimulation period. Fisher's exact test was performed to determine differences in response rate between conditions. P = 0.0093. Download Figure 4-2, DOCX file.

## Discussion

Like all animals, *C. elegans* are able to process multiple pieces of sensory information (Stein et al., 2014; Yau et al., 2015; Ghosh et al., 2017; Harris et al., 2019). During behavior, when a new cue is introduced, the neural signal from the new cue is incorporated into the nervous system, which can result in different behavioral output. We found that while food-deprived worms typically avoid the dispersal pheromone osas#9, they will no longer avoid this pheromone when it is mixed with an extract of the food (Fig. 1*B*). Osas#9 avoidance attenuation is dependent on the strength of food extract present (Fig. 1*C*), indicating that the nervous system weighs the strength of each cue when processing multiple pieces of information, and this principle is true across the animal kingdom (Stein and Stanford, 2008; Leathers and Olson, 2012).

Through our work, we found that food-dependent osas#9 avoidance attenuation requires serotonergic signaling (Fig. 2*B*). In humans, serotonin is associated with reward and punishment systems and thus plays a role in reward-related decision-making (Seymour et al., 2012). Depleted serotonin and mutations in serotonin receptors are associated with riskier decision-making (Rogers, 2011). Serotonin's role in decision-making likely works through its ability to modulate neural circuits (Celada et al., 2013; Jacob and Nienborg, 2018; Marquez and Chacron, 2020). In multiple mammal models and across multiple sensory systems, serotonin application has been shown to dampen sensory neural signals (Jacob and Nienborg, 2018; Marquez and Chacron, 2020). And because serotonin receptors can be inhibitory or excitatory, there is a variety of ways that serotonin signaling can tune a circuit (Celada et al., 2013). Our results suggest that serotonin acts in a similar neuromodulatory role in *C. elegans* when animals encounter food extract during sensation of the aversive pheromone osas#9.

We found that the inhibitory serotonin receptor MOD-1 was required for food-dependent osas#9 avoidance attenuation (Fig. 2*C*). MOD-1 is expressed in numerous interneurons and motor neurons that modulate worm movement (Harris et al., 2009; Hammarlund et al., 2018). Since MOD-1 is an inhibitory chloride channel (Ranganathan et al., 2000), we propose that during food sensation, MOD-1 is activated on avoidance-promoting neurons, repressing avoidance behavior. Since we also noticed that *mod-1* mutants seemed to avoid the osas#9 and extract mixture less strongly than the *tph-1* mutants, there is a possibility some other serotonin receptors may play smaller roles in integrating food cues into the nervous system. However, their role(s) may be too small to detect when MOD-1 is functioning, as it appears to be the main receptor. Alternatively, there is some research that suggests there could be as many as eight serotonin receptors in *C. elegans*, leaving the possibility that an uncharacterized receptor acts in the food cue integration pathway (Carre-Pierrat et al., 2006).

Moreover, we show that the ADF neurons require synaptic and peptidergic input from other neuron(s) for their response to food extract (Fig. 4*A–C*). From a screen of food-related neurons, we believe it is possible that the ASK neurons are sending input to the ADF neurons (Fig. 2*E*). However, there may be other neurons involved as our screen of neurons was not exhaustive. There are numerous receptors, including neuropeptide receptors, expressed on the ADF neurons that could be screened in the future to narrow down the nature of this signaling (Hammarlund et al., 2018; Taylor et al., 2021). Given that the ADF neurons require other neural input to respond to food extract, we suggest that although the ADF is a sensory neuron, it is functioning as an interneuron in this instance. This is consistent with some prior research that has shown that although the ADF neurons are sensory, they can sometimes function as interneurons (Guo et al., 2015).

**Figure 5.**
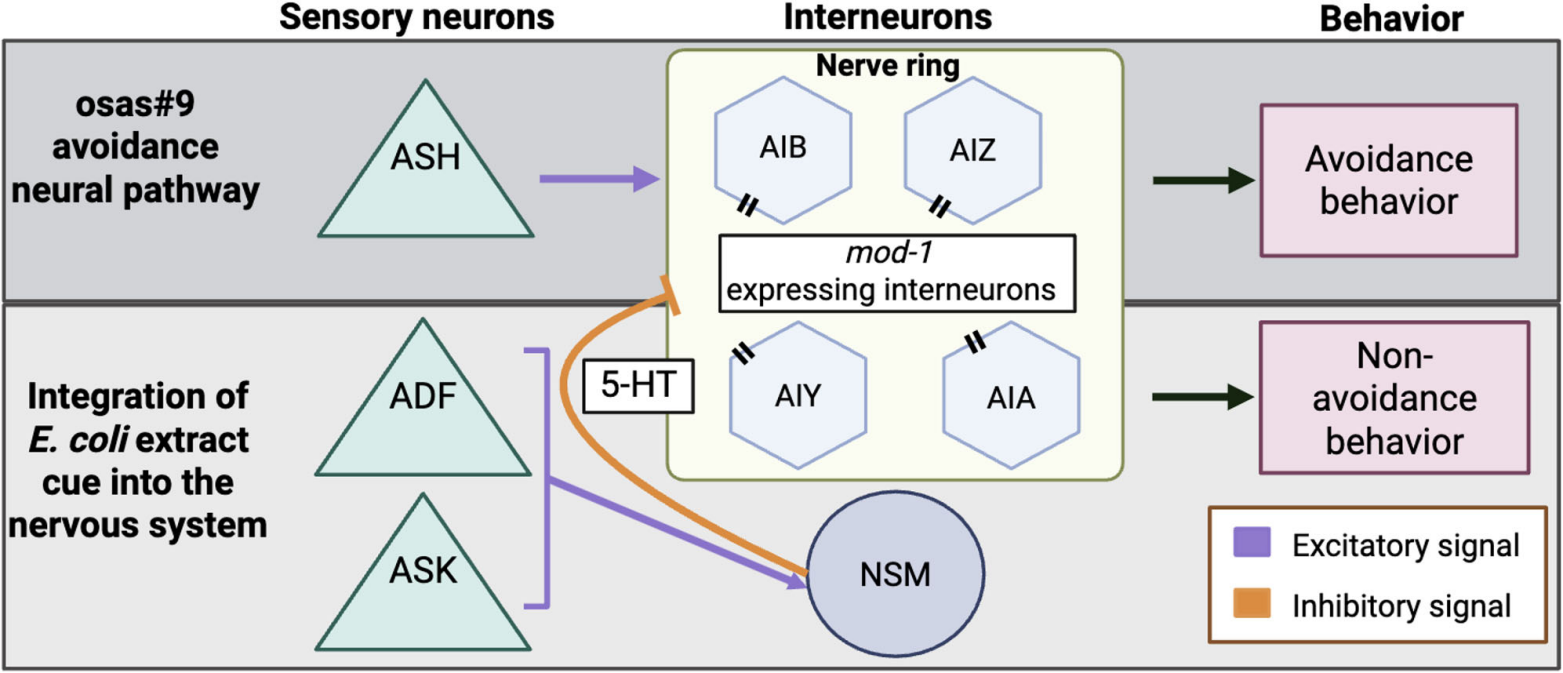
Proposed model for osas#9 avoidance attenuation. osas#9 is sensed primarily by the ASH sensory neurons (Chute et al., 2019). The ASH neurons communicate with downstream interneurons neurons to initiate avoidance. When animals are exposed to food extract, the NSM neurons send serotonergic signals that inhibit the osas#9 avoidance pathway. The serotonergic signal sent by the NSMs is likely received via MOD-1 receptors. MOD-1 receptors are expressed on interneurons that are known to modulate forward and backward motion. Created with BioRender.com.

Overall, we propose a model in which, upon sufficient *E. coli* extract sensation, an inhibitory neural pathway is activated to suppress an avoidance neural pathway (Gat et al., 2023; Liu et al., 2023; Lin et al., 2024). We suggest that the ASK and ADF function in the primary sensation of food extract. Since the ADF neurons require input from other neurons, it is possible that the ASK function upstream of the ADF. These sensory neurons probably send signals to the NSM neurons (Fig. 5). The NSM neurons are unique because they are neurosecretory (Axäng et al., 2008). It has been reported that the NSM neurons contain extrasynaptic neurosecretory terminals that secrete serotonin in the nerve ring (Jafari et al., 2011; Nelson and Colón-Ramos, 2013). We believe it is possible that this extrasynaptic serotonin binds to MOD-1 receptors expressed on movement modulating interneurons in the nerve ring, such as AIZ, AIB, AIY, and AIA (Harris et al., 2009; Hammarlund et al., 2018). Prior research has shown that ablation of the AIZ and AIB interneurons causes a reduction in reversals, implying that these interneurons promote reversal and avoidance behavior (Tsalik and Hobert, 2003; de Bono and Maricq, 2005). This type of coordinated inhibition is seen across neural circuits in the animal kingdom (Yan, 2002; Wang, 2008; Miller et al., 2011; Wu and Guo, 2011; Chadderton et al., 2014; Jacob and Nienborg, 2018; Marquez and Chacron, 2020; Herd et al., 2021). Our work adds to the body of evidence that serotonin serves an important function during multisensory processing.

## Synthesis

Reviewing Editor: James Howard, Brandeis University Decisions are customarily a result of the Reviewing Editor and the peer reviewers coming together and discussing their recommendations until a consensus is reached. When revisions are invited, a fact-based synthesis statement explaining their decision and outlining what is needed to prepare a revision will be listed below. The following reviewer(s) agreed to reveal their identity: Scott Leiser, Kevin Collins.

Summary:

In this manuscript, the authors describe a neural circuit through which C. elegans integrate positive (food extract) and negative (osas#9 pheromone) sensory cues to produce an appropriate behavioral response to this mixed-valence stimuli. Previous work has shown that pheromone osas#9 is aversive to fasted but not to fed animals. The authors build on this work by fasting C. elegans and exposing them to a mix of OP50 E. coli extract with osas#9 to test whether food chemosensation in the absence of food ingestion is sufficient to prevent osas#9 aversion. Interestingly, they find that the E. coli extract does prevent osas#9 aversion at a high enough concentration. Using a combination of behavioral assays and calcium imaging, the authors also demonstrate that this effect requires serotonin synthesis from the ADF neuron, the serotonin receptor mod-1, and likely signaling from the ASK neuron. They suggest that the ASK neuron acts upstream of ADF serotonin release because the genes unc-13 (neurotransmitter packaging) and unc-31 (neuropeptide packaging) are partially and fully required, respectively, for the osas#9/E. coli extract mixture to activate the ADF neuron. The paper thus provides evidence that serotonin signaling through the ADF neuron and MOD-1 receptor contribute to attenuation of osas#9 aversion by food perception.

Overall, the experiments generally support the model. However, the reviewers have noted a lack of the level of rigorous confirmation alongside alternative approaches that are typically expected in the C. elegant system. Some of the findings have been reported by others previously, and it is unclear whether the E. coli food extract is truly 'modulating' the avoidance response vs. just driving a strong attractive response where levels of the ascaroside cannot drive avoidance. Such a distinction may be academic, but since the authors do not show how food modulates the ascaroside response or the ascaroside modulates the food response (at the level of activity of downstream neurons, for example), evidence for the co-modulation model remains incomplete. Most experiments manipulate both factors at the same time and compare those responses to no addition. The summary model and a previous study suggests that osas#9 acts through the polymodal ASH sensory neuron, although the paper does not include much discussion of that pathway past some of the behavior avoidance experiments. The reviewers also noted a lack of attention to detail in the way some of the technical aspects of the experiments were described, although this can likely be fixed through revision. Food signals have been shown to affect ADF activity, and serotonin signaling through MOD-1 has similarly been shown to alter roaming in the enhanced slowing response. The authors make good use of tools already available to make quick progress, but the manuscript falls short of fully describing the mechanism. Specific comments and suggestions are as follows:

1. The evidence putting the ASK neuron upstream of the ADF neuron in their model is circumstantial. The authors show that the ADF neuron only responds to the osas#9/E. coli extract stimuli when neurotransmitter and neuropeptide signaling are intact. They also show that the ASK sensory neuron is required for E. coli extract attenuation of osas#9 aversion. The data also show a trend for the AWC neurons to be required, although these animals appear too variable to reach significance. From this information, they speculate that the ASK neuron signals directly to the ADF neuron following E. coli extract exposure, while they could alternatively signal in parallel or downstream (less likely). To directly test this hypothesis, the authors should measure osas#9/E. coli extract-mediated ADF activity in the ASK ablation strain. If the ADF does not respond to this stimulus when ASK is ablated, this would confirm that ASK acts upstream rather than downstream or parallel of the ADF in this circuit. Follow up on the AWC could also be interesting, but is likely challenging due to the variability in that strain.

2. Figure 1: It's not clear what the 'solvent control' sample is supposed to be. Is it the solvent for the osas#9, the E. coli extract, or both? As that control moves forward, it seems to be it is lacking osas#9 and therefore is unlikely to drive an avoidance response. Does the E. coli extract by itself do anything?

3. It may not be necessary to cite BioRender for the images showing in Fig. 1A. The amount of liquid applied to the worm seems significant, possibly even inducing changes in locomotion gait that might be associated with swimming (see Vidal-Gadea 2011). It may be worth discussing this role for serotonin and whether / how the food responses here are different and/or exclude this pathway from consideration. Would it be possible to show a movie of this behavior?

4. In the legend for Figure 1, can the authors specify if/ which data are normally vs. non-normally distributed? For example, if data in b is normally distributed (seems like it), why not just say a one-way ANOVA was used? If the data in 'C' are not normally distributed, why not just say a Kruskal-Wallis test was used? In Figure 1C, the data may make more intuitive sense if they are shown in increasing extract concentration starting from 0 rather than the other way around. Also, this data seems like it should be shown in an XY plot rather than categorical series.

5. Figure 2: the data in Figure S2A should be included here in the main Figure to show the osas#9 is able to drive an avoidance response in the mutants.

6. When describing Figure 2B in lines 180-181, the authors state that "avoidance behavior in mod-1 worms trended a little bit lower than avoidance behavior of tph-1 mutants". Given this observation, and the data in this panel showing that the ser-7 knockout has a nonsignificant trend towards phenotyping tph-1, it appears that multiple serotonin receptors may contribute to this circuit. To try and achieve a response comparable to the tph-1 knockout, the authors could consider testing a double knockout of ser-7 and mod-1.

7. It's not completely clear how the rescue experiments shown in Figure 2C were performed, as details about the strains used are not include in the Materials &Methods. There are some strains in the Table, but the legend is a bit vague. There are three strains in the Table that might be related to this experiment (CX13571, CX13572, and SWF855), and it's not clear how these strains would be mentioned elsewhere in the Table other than for this Figure. The first two strains represent different Cre driver lines allowing for cell-specific excision of a LoxP flanking tph-1 MosCI knockin. The third strain expresses tph-1 in the HSNs from the egl-6 promoter (along with some other stuff). The correct interpretation for these experiments may actually be that tph-1 is required in ADF but not NSM. The HSN experiment can only be interpreted as tph-1 expression in HSN is not sufficient to rescue. It may therefore not be required there or that it is also required somewhere else (e.g. ADF). Unless the reviewers are misinterpreting these experiments, the Methods, Figure, legend, and text should be fixed. Consider adding some of the specifics to the Table including which strains, what they were used for, and in which Figures.

8. Figure 3: Consider connecting the paired data together with lines to show each animal responded to the extract. Also, why is the osas#9 not added in this experiment? It appears the authors think that ascaroside is acting on another sensory neuron, but it might still be useful to show no (direct) differences in ADF activity in response to food. It seems this figure simply shows that ADF neurons respond to signals in food which has already been shown (Iwanir et al. 2016). Also, what is the evidence that the component of the E. coli extract is an odor? Can it act at a distance?

9. In the calcium imaging data shown in Figures 3B-C, it appears that there is a bimodal distribution in the ADF response to stimulus. The authors should explain why about half of their subjects did not respond at all to even the highest tested concentrations of E. coli extract, and if this type of variability is expected in calcium imaging data. Do the worms with minimal ADF activity in response to the stimulus behave differently than the worms with higher ADF activity following osas#9/E. coli extract exposure?

10. Also, in Figures 3 and 4, the authors discuss the phenomenon of 'no response' in ADF. The scatterplots of ADF calcium responses show that there are some responding animals and (many) non-responding animals, regardless of E. coli extract. It would be useful for the authors to find some more objective criteria than ∆R/R and 'responding vs. non-responding' to analyze whether there are in fact dose-dependent responses in ADF calcium following stimulation. This would require some kind of threshold beyond which a response is counted vs. not. Since different experiments show different peak ∆F/F and may have distinct baseline ∆F/F levels prior to stimulus, this may be challenging. However, the authors should try to analyze if there is a statistical difference in the yes/no responses seen, for example by using Fisher Exact tests, etc. Additionally, connecting the lines would probably help in the visualization of responders vs. non-responders.

11. Figure 3 legend (title): the Wilcoxon tests do not appear to definitively inform what the specific features are that are different between the two groups, just that they are different. The test does not say which one has a higher maximum fluorescence intensity, just that they are different. You would expect to see the same p value if the 'Stimulation' values were negative, for example. To measure differences, you would have to compare means or medians if that is the conclusion you wish to draw. The authors may be able to measure the increase in Ca2+ by subtracting the pre-value and then comparing peak ∆F/F across the different E. coli extract conditions to show a dose-dependent increase in ADF Ca2+.

12. It would be helpful to show a WT control in Figure 4A to allow readers to compare the blunted response in unc-13 worms to a typical ADF response.

13. Figure 4D: It's unclear if ablation results for AWC and ASK affect osas#9, E. coli extract, or both. Please include avoidance behavior responses to osas#9 alone unless these have been shown elsewhere.

14. Figure S3 heat map look-up table is not consistent among different recordings. Does variability in different experiments prevent these numbers from being consistent? Or, are the ADF calcium responses seen much lower as you decrease the E. coli food extract? Would that not also be valuable to compare? Could it be the osas#9 affects the resilience of the ADF responses such that they are more robust? Genetically, ADF is predicted to be downstream of whatever cell osas#9 is acting, but is there data (e.g. from ablations) that loss of ADF or other neurons has no effect on avoidance? The reviewers do not necessarily suggest that the authors compare all the conditions just for the sake of it, but their absence is conspicuous. At the least, the Results do not emphasize why such experiments would be superfluous.

15. The heatmaps in Figure S3 and Figure S4 should be modified so the color scale is the same across all panels in the same figure. It is difficult for the reader to compare this data when, for example, green represents 0 in some panels and green represents 300 in other panels.

16. Methods: is the max ∆F/F value detected after averaging of the cell body fluorescence across segmented object pixels? Or was it simply the single hottest pixel during the recording? It seems like it would be better to describe how the cell body was selected (e.g. using some thresholding information) and then the pixel intensity was averaged over the object with some kind of correction for size (e.g. smaller cell bodies might have higher ∆F/F changes relative to a larger cell body). How big is a typical ROI?

17. In the methods section and in the main text, the description of the avoidance assay could be improved. The methods section states, "The avoidance assay was performed by placing a small drop of stimulus on the tail of a forward moving animal...Once the liquid encountered the animal's head, the animal's response would be scored as avoidance or non-avoidance..." It is confusing how placing a drop of the stimulus on the tail of a forward-moving animal will ever reach the head of the animal. Additionally, if the droplet is placed on the tail, it is unclear why a reversal (or a movement towards the stimuli) is scored as aversive.

18. The language "olfactory stimuli" in the title and throughout the manuscript is misleading. Because both the osas#9 pheromone and the OP50 extract are dissolved in water and presented to the worm in a liquid form, it is impossible to determine whether this effect is mediated by olfactory sensation of volatiles or chemosensation via activation of receptors that bind water-soluble compounds in the food extract.

19. In many of the figures and the strain list, the tph-1 knockout allele is listed as "mg2080". The correct allele for this strain is "mg280".

20. In the model figure, Figure 5, it is confusing why the ADF neuron has both an arrow and an inhibition signal, when the text only describes ADF serotonin as inhibiting potential interneurons through mod-1 binding.

21. Line 181: than for that

22. Line 189, change phalangeal to pharyngeal

23. Line 258, the text should read "also act to integrate food signals..."

24. Line 266, the B in "Figure 1b" should be capitalized

25. Line 305, the authors should cite the previous work that identified this avoidance neural pathway.

26. The authors should cite the final research paper Taylor et al., 2021, rather than the pre-review Hammarlund et al., 2018.

## Author Response

Responses to reviewer comments Comment-1: The evidence putting the ASK neuron upstream of the ADF neuron in their model is circumstantial. The authors show that the ADF neuron only responds to the osas#9/E. coli extract stimuli when neurotransmitter and neuropeptide signaling are intact. They also show that the ASK sensory neuron is required for E. coli extract attenuation of osas#9 aversion. The data also show a trend for the AWC neurons to be required, although these animals appear too variable to reach significance. From this information, they speculate that the ASK neuron signals directly to the ADF neuron following E. coli extract exposure, while they could alternatively signal in parallel or downstream (less likely). To directly test this hypothesis, the authors should measure osas#9/E. coli extract-mediated ADF activity in the ASK ablation strain. If the ADF does not respond to this stimulus when ASK is ablated, this would confirm that ASK acts upstream rather than downstream or parallel of the ADF in this circuit. Follow up on the AWC could also be interesting, but is likely challenging due to the variability in that strain.

Author Response: We thank the reviewer for highlighting the broader sensory roles of the ASK neurons. You are correct that ASK contributes to multiple behaviors-most prominently in avoidance (secondary to ASH; Hilliard et al., 2002), dauer pheromone detection (Kim et al., 2009), and food‐searching (Gray et al., 2005; Calhoun et al., 2015). We agree that, while our ablation and imaging data clearly show ADF activity depends on intact synaptic (unc-13) and peptidergic (unc-31) signaling, they do not definitively place ASK upstream of ADF. Accordingly, we revised the manuscript to (1) acknowledge the multiple functions of ASK, (2) clarify that ASK is "necessary but not proven to be upstream" of ADF, and (3) emphasize that our data support the involvement of one or more sensory neurons-including ASK-within the upstream circuit feeding into ADF, without implying a strict linear hierarchy. These textual changes will better reflect the limitations of our model without requiring additional experiments.

Comment-2: Figure 1: It's not clear what the 'solvent control' sample is supposed to be. Is it the solvent for the osas#9, the E. coli extract, or both? As that control moves forward, it seems to be it is lacking osas#9 and therefore is unlikely to drive an avoidance response. Does the E. coli extract by itself do anything? Author Response: We agree that an explanation of the solvent control is useful. Our revised manuscript includes an explanation for the solvent control in the methods section. Additionally, we have included data in the supplementary information showing that worms do not avoid E. coli extract alone.

Comment-3: It may not be necessary to cite BioRender for the images showing in Fig. 1A. The amount of liquid applied to the worm seems significant, possibly even inducing changes in locomotion gait that might be associated with swimming (see Vidal-Gadea 2011). It may be worth discussing this role for serotonin and whether / how the food responses here are different and/or exclude this pathway from consideration. Would it be possible to show a movie of this behavior? Author Response: We thank the reviewers for bringing this oversight to our attention. We have removed the reference to BioRender in Fig. 1A. Additionally, we have added new text explaining the drop size. Finally, we have included supplemental videos showing drop size and avoidance/non-avoidance behavior.

Comment 4: In the legend for Figure 1, can the authors specify if/ which data are normally vs. non-normally distributed? For example, if data in b is normally distributed (seems like it), why not just say a one-way ANOVA was used? If the data in 'C' are not normally distributed, why not just say a Kruskal-Wallis test was used? In Figure 1C, the data may make more intuitive sense if they are shown in increasing extract concentration starting from 0 rather than the other way around. Also, this data seems like it should be shown in an XY plot rather than categorical series.

Author Response: The revised manuscript includes the figure legends with specific details for each panel. In addition, we have edited Fig. 1C to show the extract concentration in increasing order.

Comment 5: Figure 2: the data in Figure S2A should be included here in the main Figure to show the osas#9 is able to drive an avoidance response in the mutants.

Author Response: The authors thank the reviewers for this suggestion and have moved Fig. 2SA to the main text.

Comment 6: When describing Figure 2B in lines 180-181, the authors state that "avoidance behavior in mod-1 worms trended a little bit lower than avoidance behavior of tph-1 mutants". Given this observation, and the data in this panel showing that the ser-7 knockout has a nonsignificant trend towards phenotyping tph-1, it appears that multiple serotonin receptors may contribute to this circuit. To try and achieve a response comparable to the tph-1 knockout, the authors could consider testing a double knockout of ser-7 and mod-1.

Author Response: We thank the reviewer for this thoughtful suggestion. In the revised manuscript (Lines 180-181 and Discussion), we now note that, while mod-1 and ser-7 single mutants each show only modest, nonsignificant reductions in avoidance-relative to the robust block seen in tph-1-testing a combined ser-7; mod-1 double knockout would likely reveal whether these two receptors act redundantly or additively in mediating serotonin's role in this circuit. We have added the following sentence to the Results: "Although neither mod-1 nor ser-7 single mutants fully recapitulate the tph-1 phenotype (Fig. 2B), a ser-7; mod-1 double mutant would be expected to produce a more complete loss of serotonin-dependent avoidance, and we plan to pursue this in future work." This change acknowledges the potential for multiple serotonin receptors to contribute and frames the double knockout as a clear next step. updated the Discussion to highlight how combining mod-1 and ser-7 could unmask redundant or parallel receptor functions.

Comment 7: It's not completely clear how the rescue experiments shown in Figure 2C were performed, as details about the strains used are not include in the Materials &Methods. There are some strains in the Table, but the legend is a bit vague. There are three strains in the Table that might be related to this experiment (CX13571, CX13572, and SWF855), and it's not clear how these strains would be mentioned elsewhere in the Table other than for this Figure. The first two strains represent different Cre driver lines allowing for cell-specific excision of a LoxP flanking tph-1 MosCI knock-in. The third strain expresses tph-1 in the HSNs from the egl-6 promoter (along with some other stuff). The correct interpretation for these experiments may actually be that tph-1 is required in ADF but not NSM. The HSN experiment can only be interpreted as tph-1 expression in HSN is not sufficient to rescue. It may therefore not be required there or that it is also required somewhere else (e.g. ADF). Unless the reviewers are misinterpreting these experiments, the Methods, Figure, legend, and text should be fixed. Consider adding some of the specifics to the Table including which strains, what they were used for, and in which Figures.

Author Response: We thank the reviewer for pointing out this important oversight. Upon re‐examining our rescue strains (CX13571, CX13572, SWF855) and consulting the original Oakes et al. (2019) description, we realized that these lines do not constitute classical "rescue" of tph-1 in ADF or NSM, but rather Cre-mediated excision of a loxP-flanked MosSCI tph-1 and ectopic expression of tph-1 under the egl-6 promoter in HSN. Accordingly, we revised the Results and Figure 2C legend to clarify that CX13571 and CX13572 report cell-specific excision of tph-1 in ADF or NSM, and that SWF855 drives tph-1 expression only in HSN under the egl-6 promoter. Additionally, we corrected the text interpretation to state that tph-1 is required in ADF (as shown by loss upon excision in CX13571) but not rescued by HSN expression (SWF855), leaving open the possibility of additional sites of action.

These changes ensure accurate presentation of the genetic tools, the logic of our cell-specific manipulations, and the conclusions drawn from Figure 2C, and have been incorporated into the revised manuscript.

Comment 8: Figure 3: Consider connecting the paired data together with lines to show each animal responded to the extract. Also, why is the osas#9 not added in this experiment? It appears the authors think that ascaroside is acting on another sensory neuron, but it might still be useful to show no (direct) differences in ADF activity in response to food. It seems this figure simply shows that ADF neurons respond to signals in food which has already been shown (Iwanir et al. 2016). Also, what is the evidence that the component of the E. coli extract is an odor? Can it act at a distance? Author Response: The authors thank the reviewer for their feedback. Osas#9 was not added to the experiment because we have data showing that removal of the ADF neuron does not impact osas#9 avoidance, leading us to believe that the ADF neurons are only involved in extract sensation. We have added these data to the manuscript (Fig. S2B). Our experiment differs from that of Iwanir et al. (2016) in that they use a full bacterial lawn as an imaging stimulus. This bacterial lawn consists of live cells and serves as a nutritive food source, whereas the extract we are using contains no live cells and therefore lacks nutritive value. Finally, we have removed the mention of E. coli extract being an odor and simply refer to it as a cue because we directly expose the worm to it.

Comment 9: In the calcium imaging data shown in Figures 3B-C, it appears that there is a bimodal distribution in the ADF response to stimulus. The authors should explain why about half of their subjects did not respond at all to even the highest tested concentrations of E. coli extract, and if this type of variability is expected in calcium imaging data. Do the worms with minimal ADF activity in response to the stimulus behave differently than the worms with higher ADF activity following osas#9/E. coli extract exposure? Author Response: We have added text to discuss possible reasons for the ADF's bimodal and variable response to food extract.

Comment 10: Also, in Figures 3 and 4, the authors discuss the phenomenon of 'no response' in ADF. The scatterplots of ADF calcium responses show that there are some responding animals and (many) non-responding animals, regardless of E. coli extract. It would be useful for the authors to find some more objective criteria than ∆R/R and 'responding vs. non-responding' to analyze whether there are in fact dose-dependent responses in ADF calcium following stimulation. This would require some kind of threshold beyond which a response is counted vs. not. Since different experiments show different peak ∆F/F and may have distinct baseline ∆F/F levels prior to stimulus, this may be challenging. However, the authors should try to analyze if there is a statistical difference in the yes/no responses seen, for example by using Fisher Exact tests, etc. Additionally, connecting the lines would probably help in the visualization of responders vs. non-responders.

Author Response: We thank the reviewer for highlighting the need for a more rigorous, quantitative definition of "responders" in our ADF imaging assays. In the revised manuscript, we have:

1. Defined an objective response threshold as ΔF/F {greater than or equal to} 3× the baseline standard deviation, calculated for each animal over the 10 s pre‐stimulus period.

2. Added additional analysis of all ADF traces using this criterion to classify each trial as "responder" or "non-responder," and summarized the counts in new Supplemental Tables S2 (dose-response series) and S3 (genetic backgrounds). Fisher's exact tests are reported in each table legend, demonstrating a statistically significant increase in responder frequency with higher E. coli extract concentrations (p < 0.01).

3. Updated the Materials &Methods to describe our threshold calculation, responder classification, and statistical testing workflow.

These additions provide a precise and reproducible definition of "response," confirm dose-dependent recruitment of ADF at the population level, and enhance the clarity of responder versus non-responder dynamics in our presentation.

Comment 11: Figure 3 legend (title): the Wilcoxon tests do not appear to definitively inform what the specific features are that are different between the two groups, just that they are different. The test does not say which one has a higher maximum fluorescence intensity, just that they are different. You would expect to see the same p value if the 'Stimulation' values were negative, for example. To measure differences, you would have to compare means or medians if that is the conclusion you wish to draw. The authors may be able to measure the increase in Ca2+ by subtracting the pre-value and then comparing peak ∆F/F across the different E. coli extract conditions to show a dose-dependent increase in ADF Ca2+.

Author Response: We thank the reviewer for this important clarification. In the revised manuscript, we have updated Figure 3 legend to have the main title "The ADF neuron responds to food cues." We have also updated the rest of the figure legend to reflect the correct interpretation. These revisions ensure that our statistical description accurately reflects what the test can-and cannot conclude.

Comment 12. It would be helpful to show a WT control in Figure 4A to allow readers to compare the blunted response in unc-13 worms to a typical ADF response.

Author response: The revised figure and the associated text in the manuscript incorporates the WT background response, which has been added to the graph.

Comment 13: Figure 4D: It's unclear if ablation results for AWC and ASK affect osas#9, E. coli extract, or both. Please include avoidance behavior responses to osas#9 alone unless these have been shown elsewhere.

Author response: The authors agree that this is important to include. These data has been added to the manuscript (Fig. S2B).

Comment 14: Figure S3 heat map look-up table is not consistent among different recordings. Does variability in different experiments prevent these numbers from being consistent? Or, are the ADF calcium responses seen much lower as you decrease the E. coli food extract? Would that not also be valuable to compare? Could it be the osas#9 affects the resilience of the ADF responses such that they are more robust? Genetically, ADF is predicted to be downstream of whatever cell osas#9 is acting, but is there data (e.g. from ablations) that loss of ADF or other neurons has no effect on avoidance? The reviewers do not necessarily suggest that the authors compare all the conditions just for the sake of it, but their absence is conspicuous. At the least, the Results do not emphasize why such experiments would be superfluous.

Author Response: The authors apologize for the lack of clarity in our heatmap data in our original submission. The revised manuscript has all heatmaps on the same scale. The new color scheme highlights the varying levels of animal response at each concentration. We have also added a supplemental figure showing the maximum responses during stimulation at each extract concentration to show the different response magnitudes. We compared all responses to 1/2000 E. coli extract, as this is the concentration chosen for behavioral testing. We have also included data to show that loss of ADF does not result in loss of osas#9 avoidance.

Comment 15: The heatmaps in Figure S3 and Figure S4 should be modified so the color scale is the same across all panels in the same figure. It is difficult for the reader to compare this data when, for example, green represents 0 in some panels and green represents 300 in other panels.

Author response: In the revised manuscript, all heatmaps now use an identical ΔF/F color scale, facilitating direct comparison across conditions and genotypes. We have updated each figure legend to note the unified scale and ensure clarity for the reader.

Comment 16: Methods: is the max ∆F/F value detected after averaging of the cell body fluorescence across segmented object pixels? Or was it simply the single hottest pixel during the recording? It seems like it would be better to describe how the cell body was selected (e.g. using some thresholding information) and then the pixel intensity was averaged over the object with some kind of correction for size (e.g. smaller cell bodies might have higher ∆F/F changes relative to a larger cell body). How big is a typical ROI? Author Response: We thank the reviewer for bringing this ambiguity to our attention. We have edited the Methods section (Lines 117-121) to specify that the region of interest (ROI) used was the entire neuron. The neuron was morphologically identified, which was possible due to baseline fluorescence of GCaMP in the cells of interest. These details have been added to ensure full transparency of our imaging analysis workflow.

Comment 17: In the methods section and in the main text, the description of the avoidance assay could be improved. The methods section states, "The avoidance assay was performed by placing a small drop of stimulus on the tail of a forward moving animal...Once the liquid encountered the animal's head, the animal's response would be scored as avoidance or non-avoidance..." It is confusing how placing a drop of the stimulus on the tail of a forward-moving animal will ever reach the head of the animal. Additionally, if the droplet is placed on the tail, it is unclear why a reversal (or a movement towards the stimuli) is scored as aversive.

Author Response: The revised text has been updated to clarify how the avoidance assay was performed and why animals are scored as avoiding upon reversal behavior. We have also included supplemental videos showing how the assay is scored.

Comment 18: The language "olfactory stimuli" in the title and throughout the manuscript is misleading. Because both the osas#9 pheromone and the OP50 extract are dissolved in water and presented to the worm in a liquid form, it is impossible to determine whether this effect is mediated by olfactory sensation of volatiles or chemosensation via activation of receptors that bind water-soluble compounds in the food extract.

Author Response: We thank the reviewer for highlighting this vital distinction. The language in the paper and title has been edited to exclude the mention of "olfactory stimuli" since stimuli are directly applied to the animal.

Comment 19: In many of the figures and the strain list, the tph-1 knockout allele is listed as "mg2080". The correct allele for this strain is "mg280".

Author response: This has been corrected in the revised manuscript.

Comment 20: In the model figure, Figure 5, it is confusing why the ADF neuron has both an arrow and an inhibition signal, when the text only describes ADF serotonin as inhibiting potential interneurons through mod-1 binding.

Author Response: The authors acknowledge the lack of clarity in the figure and have modified Figure 5 to reflect our edits and for clarity.

Comment 21: Line 181: than for that Author Response: This typo has been corrected.

Comment 22: Line 189, change phalangeal to pharyngeal Author response: This has been corrected.

Comment 23: Line 258, the text should read "also act to integrate food signals..." Author Response: The revised text incorporates the change.

Comment 24: Line 266, the B in "Figure 1b" should be capitalized Author Response: This typo has been corrected.

Comment 25: Line 305, the authors should cite the previous work that identified this avoidance neural pathway.

Author Response: The authors have added citations acknowledging previous work in the revised manuscript.

Comment 26: The authors should cite the final research paper Taylor et al., 2021, rather than the pre-review Hammarlund et al., 2018 Author Response: The authors thank the reviewers for pointing this out. The citation has been changed.
