# Supplementary figures and images for: Serotonergic Signaling Governs Caenorhabditis elegans Sensory Response to Conflicting Chemosensory Stimuli
Source: eNeuro. 2025 Jul 17;12(7):ENEURO.0127-25.2025. doi: 10.1523/ENEURO.0127-25.2025 (PMC12303587; doi:10.1523/ENEURO.0127-25.2025)

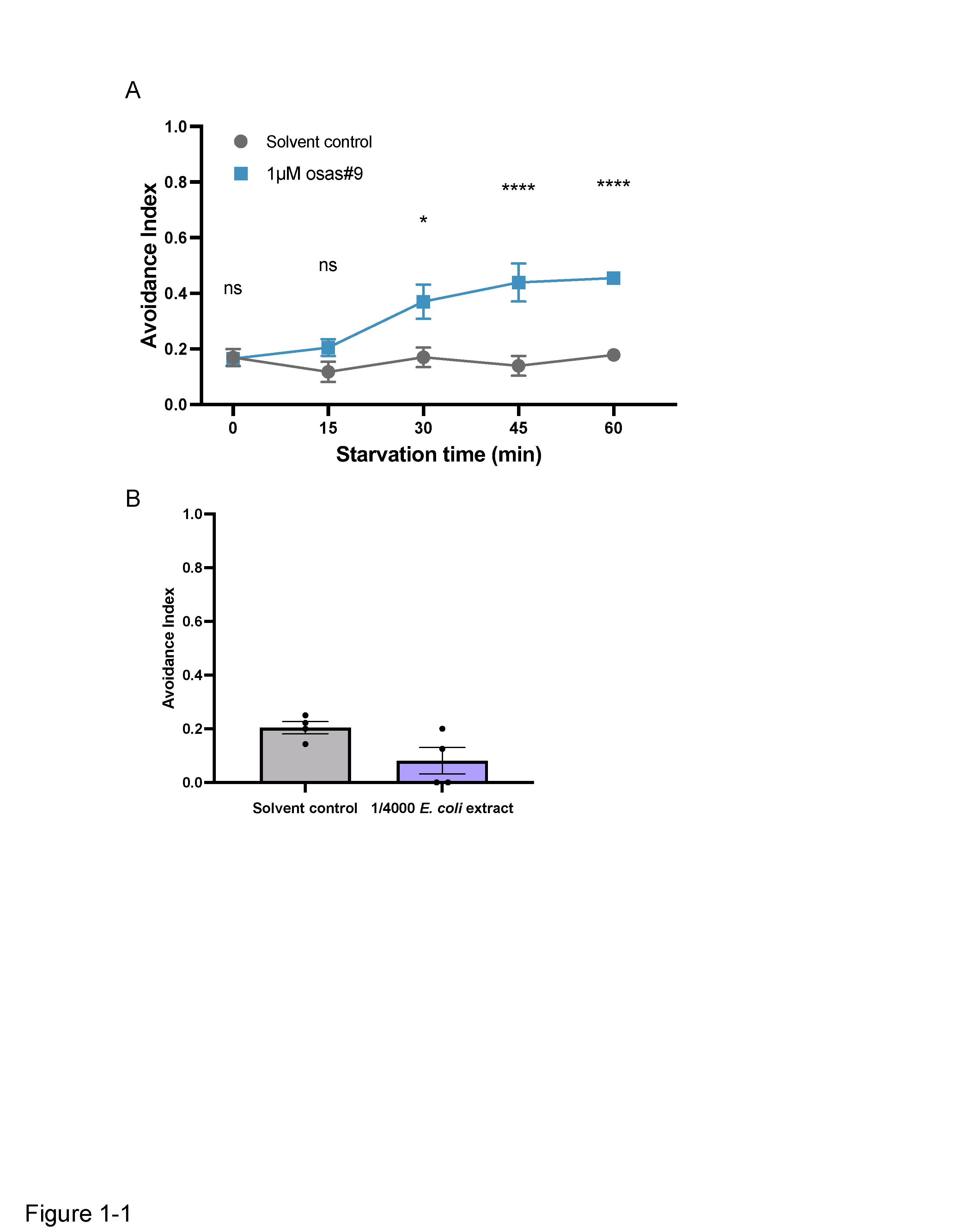

Supplement: Figure 1-1 — a. Animals will begin avoiding 1 µM osas#9 30 minutes after food removal. Prior to 30 minutes, avoidance index to 1 µM osas#9 is no different than the solvent control. The avoidance index appear to plateau after 30 minutes of food removal. n >= 7.b. Animals do not avoid E. coli extract alone. Download Figure 1-1, TIF file. [file eneuro-12-ENEURO.0127-25.2025-s004.tif]

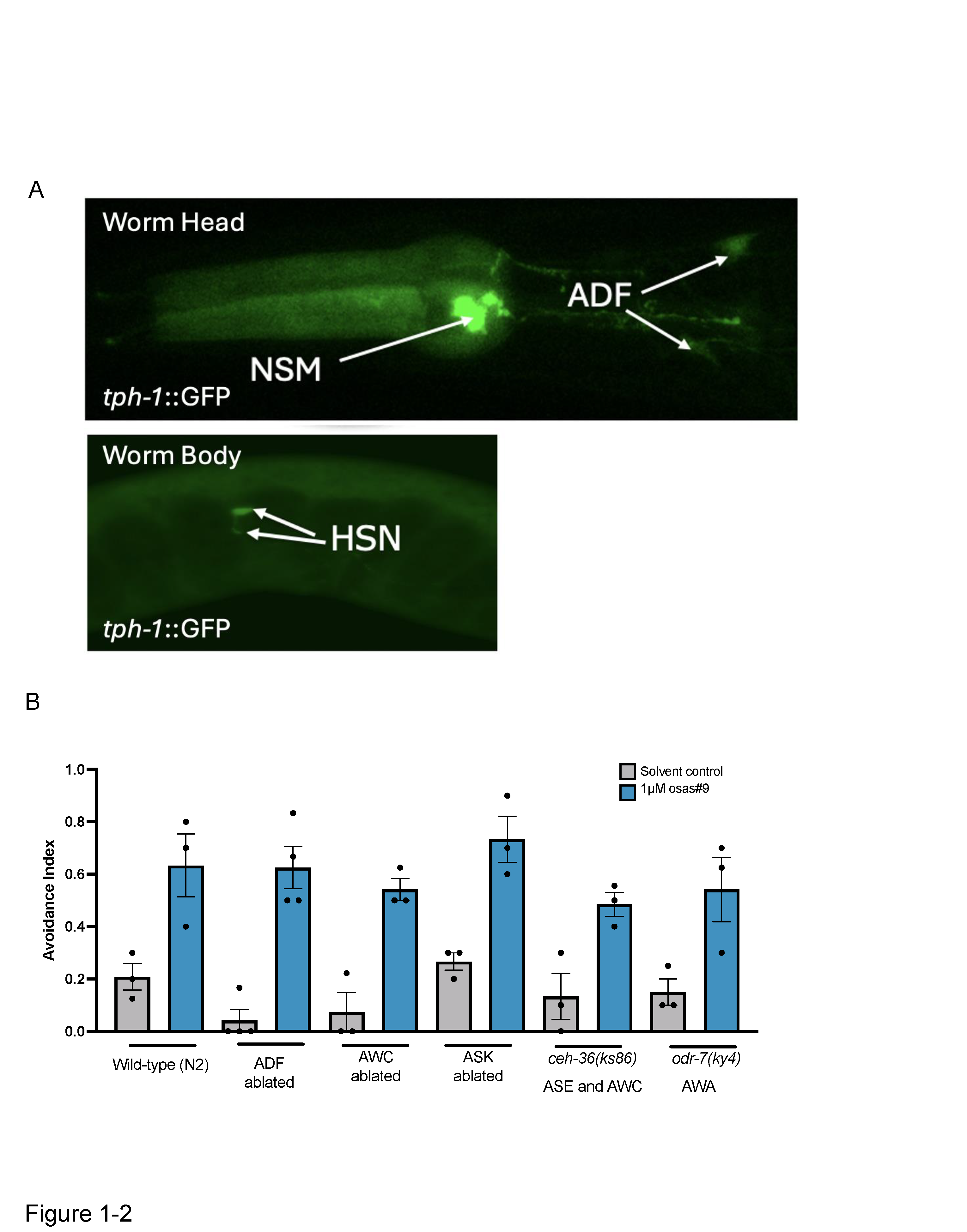

Supplement: Figure 1-2 — a. Ablation of ADF, ASK, or AWC neurons does not impact osas#9 avoidance; ceh-36 and odr-7 animals avoid osas#9.b. Animals containing tph-1::GFP constructs showed tph-1 expression in the ADF, HSN, and NSM neurons. 63X magnification; oil objective. Download Figure 1-2, TIF file. [file eneuro-12-ENEURO.0127-25.2025-s005.tif]

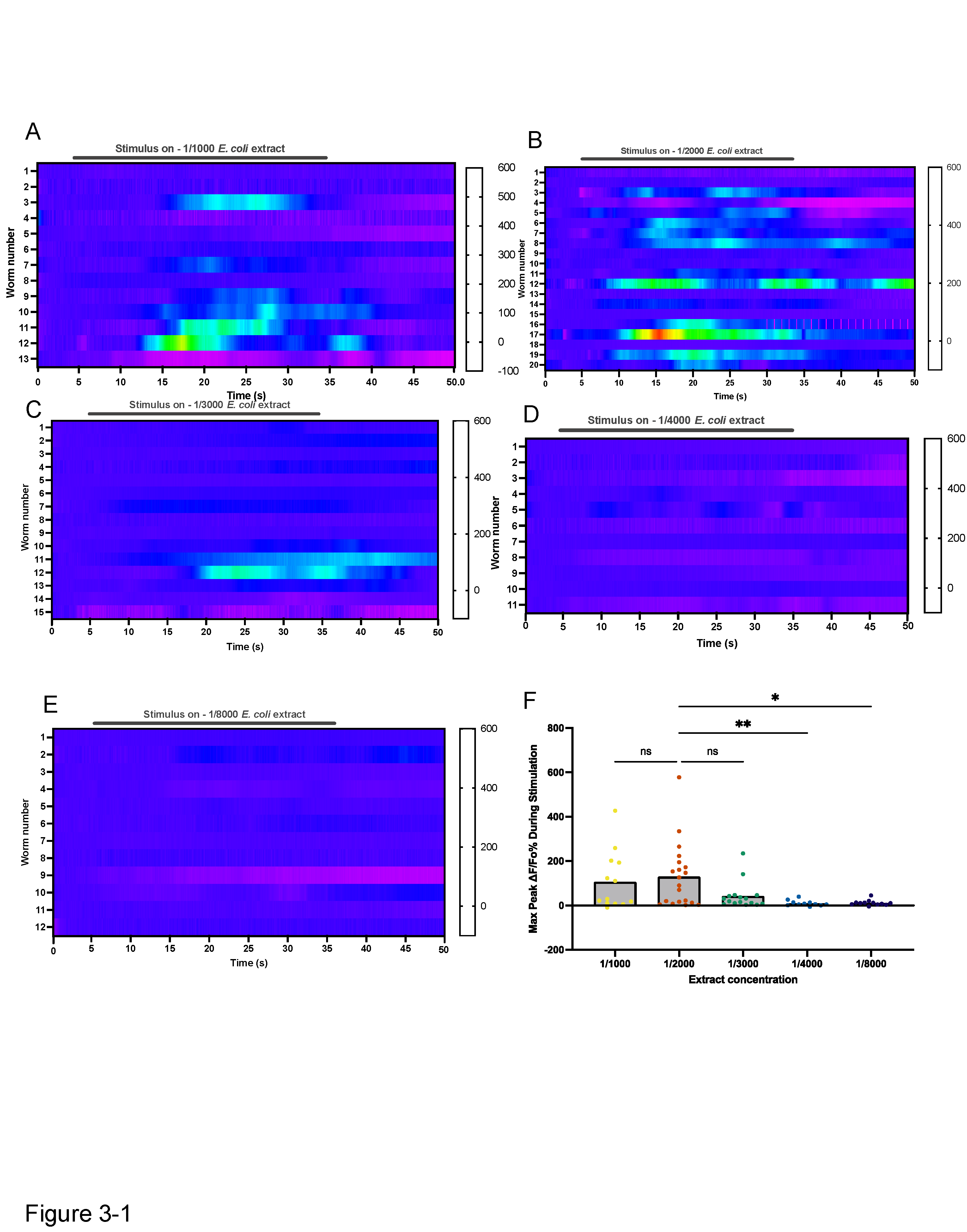

Supplement: Figure 3-1 — Heatmaps of fluorescence intensity in the ADF neurons in ADF::GCaMP animals upon exposure to a.1/1000 E. coli extract, b. 1/2000 E. coli extract, c. 1/3000 E. coli extract, d. 1/4000 E. coli extract, e. 1/8000 E. coli extract. n >=11. f. Magnitude of maximum peak response during stimulation with each extract concentration. Shapiro-wilk normalcy test followed by ordinary one-way ANOVA w/ Sidak’s multiple comparisons. All concentrations compared to 1/2000 extract because this was the concentration used for behavioral assays. *p < 0.05, **p < 0.01, ***p < 0.001, ****p < 0.0001. Download Figure 3-1, TIF file. [file eneuro-12-ENEURO.0127-25.2025-s006.tif]

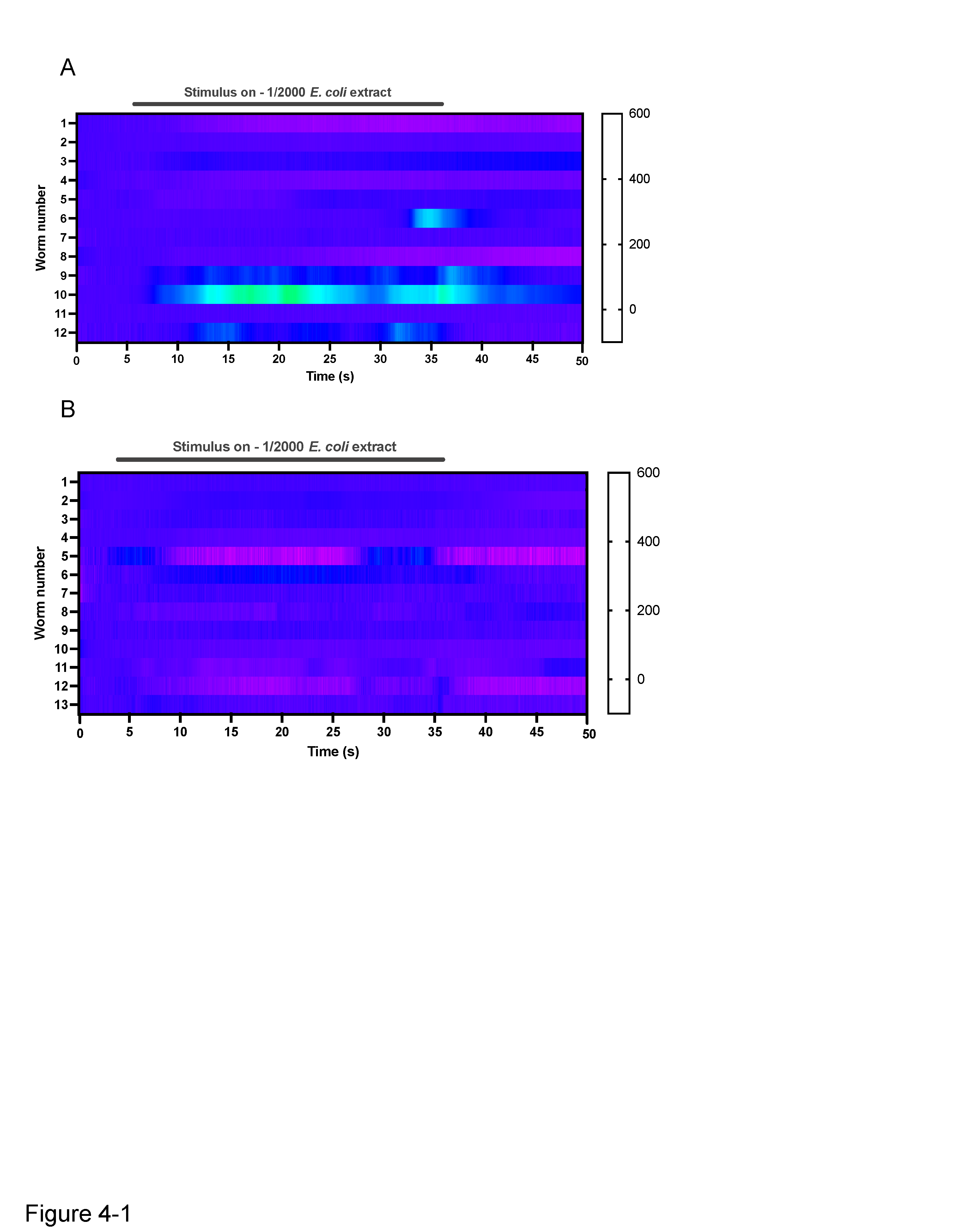

Supplement: Figure 4-1 — Heatmaps of fluorescence intensity in the ADF neuron in ADF::GCaMP animals upon exposure to 1/2000 E. coli extract in a. an unc-13(e51) background and b. an unc-31(e928) background. Download Figure 4-1, TIF file. [file eneuro-12-ENEURO.0127-25.2025-s008.tif]
