# Supplementary material for: Serotonergic Signaling Governs Caenorhabditis elegans Sensory Response to Conflicting Chemosensory Stimuli
Source: eNeuro. 2025 Jul 17;12(7):ENEURO.0127-25.2025. doi: 10.1523/ENEURO.0127-25.2025 (PMC12303587; doi:10.1523/ENEURO.0127-25.2025)
Supplement: Table 1-1 — List of strains used in this manuscript. Download Table 1-1, DOCX file. [file eneuro-12-ENEURO.0127-25.2025-s010.docx]

**Table 1-1:**

| Strain name | Genotype | Source | Figure |
| --- | --- | --- | --- |
| N2 | Wildtype | CGC | 1B, 1C, 2A-E, 1-1A,B, 1-2A, |
| MT15434 | *tph-1 (mg280)* II | CGC | 2A-D |
| CB1112 | *cat-2(e1112)* II | CGC | 2A,B |
| MT13113 | *tdc-1(n3419)* II | CGC | 2A,B |
| CX13503 | *eat-4 (ky5)* | Bargmann Lab | 2A |
| CB156 | *unc-25(e156)* III | CGC | 2A |
| DA1814 | *ser-1(ok345)* X | CGC | 2C |
| AQ866 | *ser-4(ok512)* III | CGC | 2C |
| RB2277 | *ser-5(ok3087)* I | CGC | 2C |
| RB1585 | *ser-7(ok1944)* X | CGC | 2C |
| MT9668 | *mod-1(ok103)* V | CGC | 2C |
| SWF912 | *lgc-50(flv8)* III; flvIs2[*tph-1p*(short)::Chrimson + *elt-2p*::mCherry] | Flavell Lab/CGC | 2C |
| CX13571 | *tph-1(mg280);* kySi56; kyEx4077= *srh-142*::nCre (95 ng/uL); *myo-3*::mCherry (5ng/uL) | Bargmann Lab | 2D |
| CX13572 | *tph-1(mg280);* kySi56; kyEx4057= *ceh-2*::nCre (10 ng/uL); *myo-3*::mCherry (5ng/uL) | Bargmann Lab | 2D |
| SWF855 | *tph-1(mg280)*; flvIs2[*tph-1*(NSM-specific fragment)::Chrimson, *elt-2*::mCherry]; flvEx401[*pegl-6*::*tph-1* cDNA*, pmyo-*2::mcherry] | Flavell lab | 2D |
| GR1366 | mgIs42 [tph-1::GFP + rol-6(su1006)] | CGC | 1-2B |
| NFB1445 | *rrf-3*(pk1426) II; *lite-1*(ce314) X; vlcEx1292[*srh-142p*::mCherry::SL2::GCaMP3 + *unc-122p*::RFP] | CGC | 3A-F, 3-1A-F |
| JSR182 | *unc-31 (e928);* *rrf-3(pk1426)* II; *lite-1(ce314)* X; vlcEx1292 [*srh-142p*::mCherry::SL2::GCaMP3 + *unc-122p*::RFP] | Srinivasan lab | A4,C, 4-2 |
| JSR184 | *unc-13 (e51)*; *rrf-3(pk1426)* II; *lite-1(ce314)* X; vlcEx1292 [*srh-142p*::mCherry::SL2::GCaMP3 + *unc-122p*::RFP] | Srinivasan lab | 4A,B, 4-1A |
| UR987 | *him-5(e1490)* V; udEx212[*Pelt-2*:: GFP; *Psrh-142*::GFP; *Psrh-142*::*ced-3(p15)*; *Psrh-142*::*ced-3*(p17) line 2] | Strain from Portman lab and extrachromosomal array generated in Ferkey lab: https://doi.org/10.1371/journal.pgen.1006153 | 2E, 1-2A |
| PS6022 | qrIs1[*sra-9*::mCasp1] | Sternberg lab | 2E, 1-2A |
| PY7502 | oyIs85 [*ceh-36p*::TU#813 + *ceh-36p*::TU#814 + *srtx-1p*::GFP + *unc-122p*::DsRed] | CGC | 2E, 1-2A |
| FK311 | *ceh-36(ks86)* X | CGC | 2E, 1-2A |
| CX4 | *odr-7(ky4)* X. | CGC | 2E, 1-2A |
