# Supplementary material for: Serotonergic Signaling Governs Caenorhabditis elegans Sensory Response to Conflicting Chemosensory Stimuli
Source: eNeuro. 2025 Jul 17;12(7):ENEURO.0127-25.2025. doi: 10.1523/ENEURO.0127-25.2025 (PMC12303587; doi:10.1523/ENEURO.0127-25.2025)
Supplement: Figure 3-2 — The number of responding and non-responding animals during calcium imaging of the ADF neurons using different concentrations of E. coli extract as a stimulus. An animal was counted as a responder if its maximum response during stimulation was three times or greater than its maximum response during the pre-stimulation period. Fisher's exact test was performed to determine differences in response rate between conditions. P = 0.054. Download Figure 3-2, DOCX file. [file eneuro-12-ENEURO.0127-25.2025-s007.docx]

**Figure 3-2:**

| Extract concentration | # of animals responding | # of animals not responding | Total % responding |
| --- | --- | --- | --- |
| 1/1000 | 7 | 6 | 54% |
| 1/2000 | 10 | 10 | 50% |
| 1/3000 | 10 | 6 | 63% |
| 1/4000 | 1 | 10 | 10% |
| 1/8000 | 4 | 8 | 50% |
