# Supplementary material for: Serotonergic Signaling Governs Caenorhabditis elegans Sensory Response to Conflicting Chemosensory Stimuli
Source: eNeuro. 2025 Jul 17;12(7):ENEURO.0127-25.2025. doi: 10.1523/ENEURO.0127-25.2025 (PMC12303587; doi:10.1523/ENEURO.0127-25.2025)
Supplement: Figure 4-2 — The number of unc-13 and unc-31 responding and non-responding animals during calcium imaging of the ADF neurons using 1/2000 E. coli extract as a stimulus. An animal was counted as a responder if its maximum response during stimulation was three times or greater than its maximum response during the pre-stimulation period. Fisher's exact test was performed to determine differences in response rate between conditions. P = 0.0093. Download Figure 4-2, DOCX file. [file eneuro-12-ENEURO.0127-25.2025-s009.docx]

**Figure 4-2:**

| Strain | # of animals responding | # of animals not responding | Total % responding |
| --- | --- | --- | --- |
| *unc-13* | 7 | 5 | 58% |
| *unc-31* | 1 | 13 | 8% |
